# Effect of Stress on the Work Ability of Aging American Workers: Mediating Effects of Health

**DOI:** 10.3390/ijerph16132273

**Published:** 2019-06-27

**Authors:** Tianan Yang, Taoming Liu, Run Lei, Jianwei Deng, Guoquan Xu

**Affiliations:** 1 School of Management and Economics, Beijing Institute of Technology, Beijing 100081, China; 2 Sustainable Development Research Institute for Economy and Society of Beijing, Beijing 100081, China

**Keywords:** work ability, stress, social status, aging workforces, health

## Abstract

We examined how stress affects the work ability of an aging workforce, how health mediates this relationship, and how the effects of stress on work ability differ in relation to social status. We analyzed data from the Health and Retirement Survey, namely, 2921 observations in 2010, 2289 observations in 2012, and 2276 observations in 2014. Ongoing chronic stress, social status, health status, and associations with individual work ability were assessed with ordinary least squares regression. Stress was significantly inversely associated with work ability. Health may function as a mediator between individual stress and work ability. The effects of stress and health on work ability decreased as social status increased. To cope with the challenges of aging workforces, future policy-makers should consider job resources and social status.

## 1. Introduction

In industrialized countries, including the United States, maintaining the abilities of aging workers has become a popular topic in research on the long-term health of the aging workforce [1,2]. Perceived ability to work is an individual’s sense of their capability and function in performing or satisfying the requirements of their positions and represents how well people cope with the demands of their job [3,4,5,6]. The most frequently discussed determinants of perceived ability to work are job stress and health, which can be explained by the Job Demands–Resources model (JD-R) [7,8,9,10,11]. This model assumes that job demands and job resources affect the well-being of aging workers by means of motivational and health-impairment processes and explains why reported work ability is lower among aging workers than among their younger colleagues.

Aging workers are less productive because they have less job resources to manage their job demands and because they experience cognitive changes and declines in their physiological and physical abilities [12,13,14,15,16]. Aging is related to decreases and changes in several physical functions [14,15,16] and reduces the ability to maintain homeostasis, because of reductions in processing speed, working memory, and selective attention [17,18]. It decreases resources available to cope with decreased physical energy, high workloads [19], and supervisor expectations. These job demands may increase stress and impair worker health, engagement and perceived ability to continue working [2,19,20]. Tuomi et al. found that physical and physiological capacity at age 60 years is only 60% of that at age 20 years [11]. This is attributable to the age-related decrease in the efficiency of the oxygen transport system, which is caused by decreases in maximum heart rate, stroke volume and arteriovenous oxygen difference [21]. In addition, aging is associated with changes in the circulatory system that decrease blood flow to organs and the contractile capacity of the heart and increase systolic and diastolic blood pressures [22].

If we extend the JD-R model, health, as a personal resource, can be considered an important mediator between job stress and work ability [23,24]. In the health-impairment process, job demands are strongly associated with job stress and thus impair employee health. In contrast, job resources, such as personal resources in the motivational process, are strongly associated with motivational outcomes such as perceived work ability. Personal resources such as health [18] can enhance employee resiliency and perceived ability and, by enabling successful control of their work environment, help workers achieve positive health outcomes in the future. Airila and colleagues reported that health, defined as a resource in everyday life, significantly enhanced employee work ability as part of the motivational process explained by the JD-R model and Conservation of Resources theory. Specifically, as age increases, age-sensitive losses (e.g., in physical fitness, health, sensory abilities, and basic cognitive functions) tend to outweigh resource gains (e.g., in knowledge, experience, and social status), and the resources of aging workforces, such as physical fitness, health, sensory acuity, multitasking ability, and functional brain efficacy, decrease throughout adulthood [4,25,26,27].

Most previous empirical evidence was collected in cross-sectional studies [9,28] and therefore may not illustrate trends in work ability and cannot identify causal relations among investigated variables. In addition, the role of health-related resources in the JD-R model, particularly with respect to the health impairment process and motivational process [29,30], has seldom been investigated. Therefore, we examined the causal relationships that explain how stress affects work ability in an aging workforce, how health mediates this association, and how the effects of stress on work ability differ in relation to social status.

## 2. Methods and Materials

### 2.1. Sample

We conducted a secondary analysis of data from the 2010 through 2014 waves of the Health and Retirement Survey (HRS) in the United States. The HRS measures health, retirement, and psychosocial factors and work ability of aging workers. The survey was funded by the National Institute of Aging and the Social Security Administration of the United States. The HRS was initiated in 1992 and used multistage area probability sampling to recruit adults older than 50 years for participation in biennial surveys. According to the description of the HRS, survey data were collected by face-to-face or phone interviews every 2 years. The sample population was divided into two groups, which were alternately surveyed. In other words, if subgroup 1 was surveyed at year t, subgroup 2 was surveyed at year t + 2, while subgroup 1 was surveyed again at year t + 4. To avoid the aging problem and a decrease in the number of participants over time, new samples were added every 6 years [21,22]. The variables of interest were mainly collected from a participant lifestyle questionnaire (PLQ), including the Perceived Ability to Work Scale (PAWS), stress scale, and health subjective rating [23]. Using these longitudinal data, we examined empirically the effects of ongoing chronic stressors, social status and health status on individual work ability. Detailed information on the study population and research design have been published elsewhere [24].

### 2.2. Data Manipulation

Because data for some of the target variables were not available in 2006 and 2008, we only analyzed data from 2010 through 2014 in the present longitudinal study. We then examined data quality before conducting the statistical analysis. The expectation–maximization method was used to address the problem of missing values.

**Figure 1:**
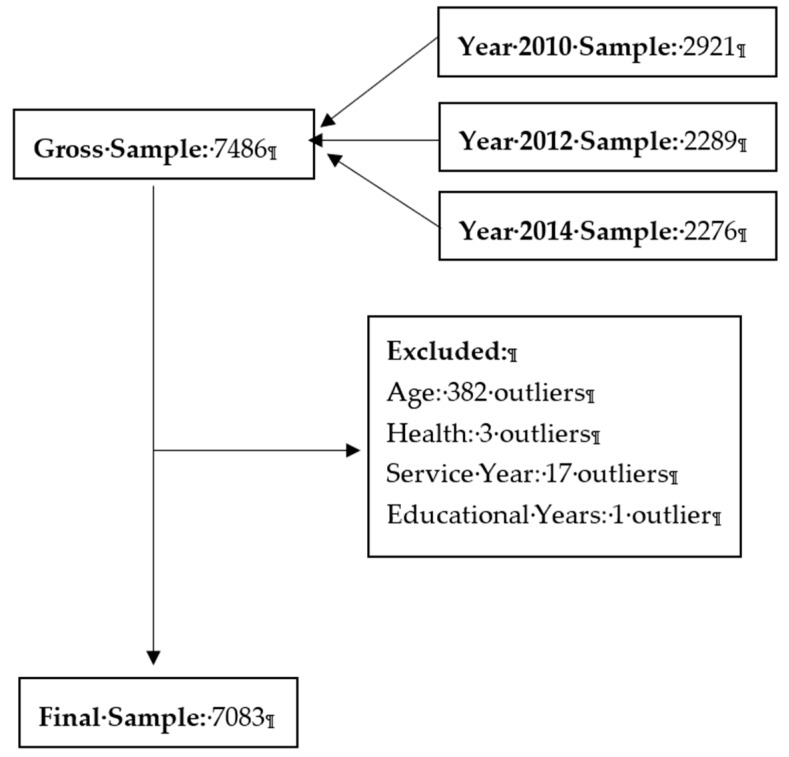
Framework for dataset generation.

After imputation, the unbalanced dataset obtained contained 7486 observations: 2921 observations for 2010, 2289 observations for 2012, and 2276 observations for 2014. Next, the final data for analysis were generated by deleting observations with unreasonable values for one or more variables. The process is shown in Figure 1.

The minimum age of HRS survey participants was 50 years; thus, the 382 observations from participants younger than 50 years were deleted. Second, three additional observations were deleted because the recorded values for the variable proxying health were outside the defined range. Third, we examined the values for control variables to correctly capture individual variation in characteristics potentially associated with work ability. Seventeen observations were deleted because they specified a year starting current position later than the survey year. One observation indicating 99 years of education was also deleted. Ultimately, a dataset of 7083 observations was used in the statistical analysis.

### 2.3. Definitions of Variables

Table 1 shows the definitions of all variables. WORK, the dependent variable, refers to work ability and measures an individual’s perceived ability to work. It was measured using PAWS because that instrument has been validated as a robust indicator of perceived productivity loss [18]. PAWS is a reliable and valid instrument and has acceptable psychometric properties [5]. The Cronbach α coefficient for PAWS was 0.89 [23] in both the HRS Psychosocial Working Group and the present study. The PAWS consists of four items, e.g., “How many points would you give your current ability to work?” (Table 2). Each item is rated from 0 (cannot currently work at all) to 10 (work ability is currently at its lifetime best). Higher values for work ability score represent greater work ability. We used the total score of the four questions. STRESS refers to stress and was measured using the six items of the “Ongoing Chronic Stressors” [25], e.g., “Ongoing difficulties at work”. Each item was rated on a four-point scale (1 = No, did not happen; 2 = Yes, but not upsetting; 3 = Yes, somewhat upsetting; 4 = Yes, very upsetting). Higher values reflect greater stress. The Cronbach α for this scale was 0.64–0.71 for the HRS Psychosocial Working Group [23] and 0.73 for the present study. This instrument has acceptable psychometric properties [25]. In this study, the logarithm of the total score for the eight questions on stress was used to investigate the association between ongoing stress and work ability. People differ in their perception of their social status, which in turn affects their work ability [26,31,32]. To examine the effect of perceived social status on work ability, SOCIAL was constructed by using the score of the PLQ question to measure subjective social status. Because our study focuses on the work ability of older workers, health status is more likely to be related to work ability [18]. Thus, we used the HEALTH from the HRS question (“Would you say your health is excellent, very good, good, fair, or poor?”) to investigate the association with work ability.

To capture differences in work ability caused by other personal characteristics, we included controls categorized into two groups. The first group was related to demographic characteristics. An individual’s ability to meet the physical needs of a job may diminish with advancing age [2]. Therefore, AGE was constructed to measure the logarithm of the respondent’s age. We calculated respondent age by subtracting the year they responded to the survey by the year of their birth. For gender, although there was no significant difference in work ability between male and female workers [27,28], we included GENDER as a control, because the association might differ in relation to age group. The variable GENDER classifies males and females and was constructed to indicate sex differences in multivariate analysis. The second control group was related to occupational characteristics. A longitudinal study found that a decrease in the work ability of aging workers was related to a “reduced working hours” policy [29]. To control for such a difference, we constructed the variable WORKLOAD by classifying original working hours into five levels. We did this because of the presence of extreme values in the dataset. WORKLOAD defines five levels of workload based on working hours per week, without the need to delete or minorize the data. An aging worker with more work experience in a position might have greater work ability [30,33]. Therefore, EXPERIENCE, i.e., the logarithm of the difference between the year respondents started their current job and the survey year, was used in our analysis. Because educational background also affects an individual’s work ability [34], the variable EDUCATION (i.e., total years of education received by an individual) was used as a proxy of educational background [35].

### 2.4. Method

**Figure 2:**
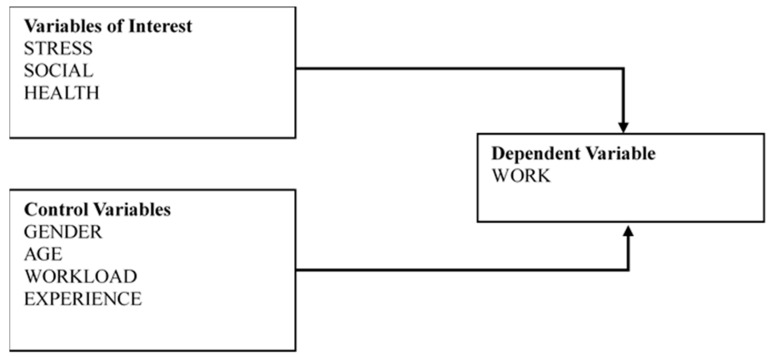
Empirical design.

As shown in Figure 2, we present an empirical model that uses ordinary least squares regression to evaluate ongoing chronic stress (STRESS), social status (SOCIAL), health status (HEALTH), and associations with individual work ability (WORK). The model is used to examine the effects of variables of interest on work ability, after controlling for variables previously identified as potential confounders in the analysis of work ability. The regression analysis of work ability is mathematically expressed below.

The subscript *it* is associated with individual *i* in year *t*. Because we collected longitudinal data for our model, we calculate heteroscedasticity-robust standard errors for our fixed effects regression model. The year fixed effects are included to capture other variation, such as job market changes over time, which affects work ability.

**Figure 3:**
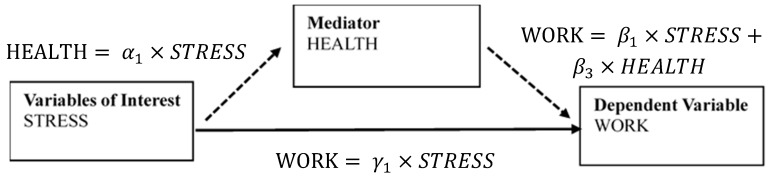
Mediation model design.

To examine the mediation effects of HEALTH [36], we designed a path model, as shown in Figure 3. Along with Equation (1), we used Equations (2) and (3) to examine mediation effects.

(1) $$\begin{array}{l} WORK_{it}=\beta_{0}+\beta_{1}STRESS_{it}+\beta_{2}SOCIAL_{it}+\beta_{3}HEALTH_{it}+\beta_{4}GENDER_{it}+\\ \beta_{5}AGE_{it}+\beta_{6}WORKLOAD_{it}+\beta_{7}EXPERIENCE_{it}+\beta_{8}EDUCATION_{it}+\mu_{it\;} \end{array}$$

(2) $$\begin{array}{ll} WORK_{it}=\gamma_{0} & +\;\gamma_{1}STRESS_{it}+\gamma_{2}SOCIAL_{it}+\;\gamma_{3}GENDER_{it}+\gamma_{4}AGE_{it}\\ & +\;\gamma_{5}WORKLOAD_{it}+\gamma_{6}EXPERIENCE_{it}+\gamma_{7}EDUCATION_{it}\\ & +\;\epsilon_{it} \end{array}$$

(3) $$\begin{array}{ll} HEALTH_{it}= & \alpha_{0}+\alpha_{1}STRESS_{it}+\alpha_{2}SOCIAL_{it}+\alpha_{3}GENDER_{it}+\alpha_{4}AGE_{it}\\ & +\alpha_{5}WORKLOAD_{it}+\alpha_{5}EXPERIENCE_{it}+\alpha_{5}EDUCATION_{it}\\ & +\theta_{it\;} \end{array}$$

First, we ran the regression according to Equation (2) to yield the coefficient $\gamma_{1}$ between STRESS and WORK. Second, we determined the significance of $\gamma_{1}$. If $\gamma_{1}$ is not significant, no mediation effect is present. Otherwise, we ran a regression according to Equation (3) to yield the coefficient, $\alpha_{1}$, between STRESS and WORK. The third step was to determine the significance of $\alpha_{1}$ in Equation (3) and $\beta_{3}$ in Equation (1). If at least one was not significant, we used the Sobel test to identify mediation effects. If both were significant, we examined whether HEALTH was a partial or full mediator, by examining coefficient $\beta_{1}$ in Equation (1). A significant $\beta_{1}$ indicates partial mediation, and a nonsignificant $\beta_{1}$ indicates full mediation.

These methods yield the total effect measured by $\gamma_{1}$, the natural direct effect measured by $\beta_{1}$, and the natural indirect effect measured by the product of $\alpha_{1}$ and $\beta_{3}$.

## 3. Empirical Results

### 3.1. Descriptive Statistics

Demographic information was missing for a few participants (0.9% to 8.7% of the overall population). Table 2 shows the descriptive statistics for all variables used in the model. For the variables STRESS, AGE, and EXPERIENCE, we used the logarithm of the original values, to improve normality for regression purpose, but report raw values here. The actual value for WORK ranged from 0 to 40. The average score, 34.57, illustrates the high work ability of respondents.

We observed heterogeneity of variables of interest in the sample. The original score for ongoing chronic stressors (STRESS) ranged from 8 to 32 in the sample, with an average of 12.54 and a standard deviation (SD) of 3.85. Similarly, the average score for SOCIAL was 6.46 (SD 1.59). The average value for health was 2.51, and the SD was even larger. Among the controls, 83% of respondents were female. The actual range for AGE was 50 to 99. As for WORKLOAD, most respondents worked full time, and one quarter worked more than 40 hours per week. Regarding EXPERIENCE, the actual value ranged from 0 to 83 years of experience in the current job; the average was 20 years (SD 14.57). Regarding EDUCATION, the respondents received 13.65 years of education on average, and the range was 0 to 17 years.

### 3.2. Correlation Matrix

Table 3 shows the correlation matrix for the variables. The lower left section shows Spearman correlation coefficients and the upper right section shows Pearson correlation coefficients. The correlations of work ability (WORK) with variables of interest were generally higher than those for other variables, indicating potential associations between dependent and independent variables. The correlations among other variables were much lower, except for those between WORKLOAD and AGE as well as between HEALTH and EDUCATION. Multicollinearity does not appear to be a significant issue, since the largest correlation is 0.343. Multicollinearity was confirmed with variance inflation factors, the highest of which was less than 2.

### 3.3. Regression Analysis

Table 4 shows the results of our main regression model. We regress WORK on STRESS, SOCIAL, HEALTH, and other control variables. As shown in column 1, the controls explained no more than 5% of the variation in work ability (WORK). After including the variables of interest, R^2^ increased to 19.30%, illustrating the statistical significance of explanatory variables. Autocorrelation was checked with the Durbin–Watson Test. The value was about 2, which indicates that autocorrelation is not a concern.

The coefficient of STRESS (*β* = −0.1043, *p* < 0.01) was significantly negatively associated with work ability (WORK); a one percent increase in STRESS decreased work ability score by about 0.0342 points. The coefficient of social status (SOCIAL) was significantly positively associated with WORK; a one-point increase in SOCIAL increased work ability by 0.42 points. The positive coefficient for the third variable of interest indicates that a one-point increase in HEALTH increased work ability (WORK) by 1.27 points.

With respect to the control variables, the negative coefficient for gender showed that, after age 59 years, work ability was lower among men than among women. The coefficient between WORKLOAD and work ability (WORK) was positive and statistically significant, which suggests that a person able to work more hours per week has greater work ability. The significant positive coefficient of EDUCATION confirmed the findings of prior studies, which reported that education improves work ability. However, the coefficient between experience (EXPERIENCE) and work ability was not significant.

### 3.4. Mediation Analysis

Health status (HEALTH) may function as a mediator between stress and work ability [37]. In accordance with prior studies, we conducted additional statistical analysis to identify potential mediation effects. Using previously described procedures, we ran the regression model in Equation (2), the results of which are shown in column 3 of Table 4. The coefficient of STRESS on WORK, $\gamma_{1}$, was significant. Therefore, we ran the regression model in Equation (3), which yielded a significant coefficient of STRESS on HEALTH, $\alpha_{1}$, as shown in column 4 of Table 4. The coefficient of HEALTH on WORK, $\beta_{3}$, was also significant, as was the coefficient for STRESS on WORK, $\beta_{1}$, which confirmed the presence of partial mediation effects. The total effects—i.e., direct effects plus indirect effects—are shown in Table 5; 24.5% of the total effect was mediated by HEALTH.

When a person has high social status, he/she may have more money, receive more education, and obtain a better job, among other advantages. Therefore, he/she may be more capable of meeting job demands. Besides, since they may have the best jobs and more resources, the stress may not affect an individual’s work ability in the same way. To examine such differences in the effects of stress, the sample was divided into three groups based on scores for subjective social status (SOCIAL). Low social status was defined as a score of 3 or lower, high social status as a score of 8 or higher, and moderate social status as a score of 4 to 7. The results of this subgroup analysis are shown in Table 6. Columns 1 to 3 list the coefficients for low, moderate, and high social status. The effects of stress on work ability decreased as social status increased. The coefficient for STRESS was −6.11 for the low social status group and only −2.90 for the high social status group, which suggests that stress has greater effect on work ability when social status is low. Similarly, people with relatively low social status have less job resources to assist them with job demands. Work therefore requires greater attention and energy, which is harmful to their health. The effects on health also decreased with increasing social status, as indicated by JDR model and the decrease in the coefficient for HEALTH from 2.47 to 0.96. In sum, the work performance of workers with low social status was more vulnerable to STRESS and HEALTH.

As shown in Table 7, we also examined the mediation effects among the groups with different social status by using the procedures of mediation effects analysis. Because all related coefficients are significant for each group, HEALTH is a partial mediator for all groups. However, by examining the percent of total effect that is mediated, we find that HEALTH mediates more of the total effects for groups with lower social status.

### 3.5. Robustness Check

As a robustness check, we replaced WORKLOAD—a variable with five levels for workload per week—with the natural logarithm of working hours per week in our main regression. The results (not tabulated) were qualitatively and quantitatively similar to those in our main analysis.

We analyzed longitudinal data in this study; thus, STRESS may have been endogenously determined as a result of reverse causality. While we found that the ratio of STRESS was associated with diminished work ability, a person with lower work ability may be more likely to have greater stress. To test the robustness of our results, we regressed WORK on the lagged variables STRESS, SOCIAL and HEALTH. LAGWORK was also included, as there may be some “stickiness” in individual work ability. As shown in Table 8, the results were consistent with those shown in column 2 of Table 4. The coefficient for LAGWORK was significant and positive, illustrating the baseline trend in work ability, while the coefficients for LAGSOCIAL became nonsignificant. The significant results for the lagged terms STRESS and HEALTH suggest that the effects of stress and health on work ability persist over time.

## 4. Discussion

In this study, chronic stress, social status, health status, and associations with individual work ability were assessed with ordinary least squares regression. Analysis of the longitudinal data showed that stress was persistently significantly inversely associated with work ability. Health mediated the relationship between individual stress and work ability, and the effects of stress and health on work ability decreased as social status increased.

Our first contribution was to use longitudinal empirical data to examine the causal relationship between stress, health and work ability and the mediating effects of health. As was the case in previous studies [38,39], stress was significantly negatively associated with work ability (WORK) in this longitudinal study, which supports a consistent effect of stress on work ability. In addition, heath was significantly positively associated with work ability. A one unit increase in health score improved work ability by 1.27 points. Ultimately, health mediated the relationship between stress and work ability. Extension of the JD-R model [38] to the health-impairment process suggests that health helps employees cope with stress at the workplace and motivates their perceived ability as part of a motivational process. Health—as a type of job resource—mediates the effects of stress on work ability because it allows workers to satisfy the demands of work and employers at workplaces. The mediating role of health and its relative resources in the JD-R model, from the perspectives of the health-impairment and motivational process, has been thoroughly investigated in longitudinal empirical studies and should be considered in future research and practice.

Our second contribution is to provide empirical evidence regarding the impact of social status on work ability. The coefficient of social status (SOCIAL) was significantly positively associated with work ability, which suggests that higher self-perceived social status improves work ability. Semi-elasticity showed that a one-point increase in social status score improved work ability by 0.42 points (average work ability score increased from 34.57 to 34.99). This result is consistent with the findings of Demakakos et al. [31] and Singh-Manoux et al. [32]. The JD-R model helps explain the effects of social status. Work conditions can be divided into job demands and job resources. Job demands require sustained physical and/or psychological effort or skills. Job resources reduce job demands and the associated physiological and psychological costs; stimulate personal growth, learning, and development; and help workers achieve work goals [40,41]. A person with high social status may have more resources, such as more money, a high level of education and a better job, among other benefits [31,32,34,42,43]. Such persons may therefore be more capable of meeting job demands. In addition, because they may have the best jobs and more resources, stress may not affect their work ability in the same way. Rizzuto and colleagues (2012) reported that individuals with higher educational attainment and those involved in highly complex and challenging jobs seemed to be more resilient. These characteristics were more common among persons with high social status [44].

Our third contribution was to confirm the effects of control variables on work ability. First, as in previous studies [2,29,34], age was significantly negatively associated with work ability, while education and workload were significantly positively associated. This is plausible because as workers age they may feel less capable of meeting the physical demands of a specific position. These older workers might have greater difficulties accepting or learning new skills, because of rapid economic or technological development [3]. In addition, aging workers with more years of education are able to handle a greater workload, which suggests that they have more social and health-related resources to cope with their job demands. To explain why the finding that EXPIERIENCE was not significantly associated with work ability is not consistent with previous studies [30,33], two plausible causes were given: On the one hand, although work experience in the current job varied greatly in the sample, it was difficult to determine if a person had performed similar jobs before, which could diminish the effect of current experience. On the other hand, older workers may have been assigned to positions that do not require extensive experience, which in turn decreased the effects of experience.

Our findings imply that further attention to health, stress and other psychosocial factors is of considerable importance in enhancing the performance of aging workers and in closing the gap between workforce supply and demand. Managers must acknowledge the central role of health among aging workforces, identify the true stressors and internal mechanisms by which stress impairs worker health and work ability and control these risk factors as part of the policy making process. For instance, to increase the productivity of an enterprise, policies must consider how to improve worker health and control the adverse effects of work–family imbalance and related psychosocial factors on health and work ability among aging workers, particular those of low social status and low workload. Then, specific interventions can be developed and implemented to help workers effectively cope with these stressors and to promote health in organizations.

This study has four limitations. First, because the data used were secondary, we were unable to collect information on some important variables of interest. Second, some respondents died of illnesses or other conditions, which resulted in survival bias in our study. Third, our use of self-reported questionnaires rather than quantitative measures limits the generalizability of our conclusions. Finally, the use of log-transformed values in the analysis might limit the generalizability of our conclusions.

## 5. Conclusions

Aging workers have less job resources and extremely high job demands, which resulted in high levels of stress. In this longitudinal study, we noted a persistent significantly negative relationship between stress and work ability and that this relationship was significantly mediated by health status, which was relatively poor among aging workers. Finally, stress had a weaker effect on the work ability of aging workers with high social status.

## Figures and Tables

**Table 1:** Definitions of variables.

Variable	Definition
WORK	The total score of 4 questions in the HRS measuring perceived work ability. Each question was scored from 0 to 10 with respect to a job’s separate general, physical, mental, and interpersonal demands. High scores indicate high work ability.
STRESS	The natural logarithm of the total score for 8 ongoing chronic stressors in the HRS survey. The score ranges from 1 to 4 for each question, and illustrates various stresses with respect to ongoing health issues of the respondent, physical or emotional problems in spouses or children, problems with alcohol or drug use in a family member, difficulties at work, financial strain, housing problems, relationship problems, and helping sick, limited, or frail family members or friends. High scores indicate high stress.
SOCIAL	Social status, as perceived by the individual. High scores indicate high self-perceived social status.
HEALTH	Health status of an individual in the survey year. The original score ranges from 1 to 5, with lower values indicating better health status. We subtracted the original values from 5, to make them more readable in the regression results. Higher scores thus indicate better health status.
GENDER	An indicator variable of the gender of an individual. Originally, 1 represented male and 2 represented female. We replaced the value of 2 with 0. Thus, 1 indicates male; other values indicate female.
AGE	The natural logarithm of the age of an individual.
WORKLOAD	An indicator variable that controls for differences in the workload of an individual. The classification process is as follows; if the original work hours per week is lower than 10, the value is 1; if 10 ≤ work hours ≤ 20, the value is 2; if 20 < work hours ≤ 30, the value is 3; if 30 < work hours ≤ 40, the value is 4; if work hours > 40, the value is 5.
EXPERIENCE	The natural logarithm of the respondent’s years of service in a job. Years of service was calculated as the natural logarithm of the difference between the year the respondent started the current job and the survey year.
EDUCATION	The total number of years of education an individual has received.

**Table 2:** Descriptive Statistics of Variables.

Variable *	*N* (%)	Mean	SD	Min	25%	75%	Max
GENDER							
Women	5880(83.0)						
Men	1203(17.0)						
WORKLOAD							
<10 h/week	268(3.8)						
10–20 h/week	490(6.9)						
20–30 h/week	860(12.1)						
30–40 h/week	3728(52.6)						
>40 h/week	1737(24.5)						
WORK		34.57	5.29	0.00	32.00	39.00	40.00
STRESS		12.54	3.85	8.00	10.00	15.00	32.00
SOCIAL		6.46	1.59	1.00	5.00	8.00	10.00
HEALTH		2.51	0.95	0.00	2.00	3.00	4.00
AGE		60.69	7.36	50.00	55.00	65.00	99.00
EXPERIENCE		20.21	14.47	0.00	7.00	32.00	83.00
EDUYEARS		13.65	2.76	0.00	12.00	16.00	17.00

* See Table 1 for variable definitions.

**Table 3:** Correlation coefficients for variables.

Variable	1	2	3	4	5	6	7	8	9
1. WORK	1	−0.288 **	0.248 ***	0.343 ***	−0.051 ***	−0.102 ***	0.133 ***	0.020 *	0.152 ***
2. STRESS	−0.257 ***	1	−0.296 ***	−0.304 ***	−0.071 ***	−0.105 ***	0.015	−0.076 ***	−0.040 ***
3. SOCIAL	0.224 ***	−0.281 ***	1	0.278 ***	0.059 ***	0.124 ***	0.056 ***	0.140 ***	0.267 ***
4. HEALTH	0.320 ***	−0.300 ***	0.269 ***	1	−0.013	−0.027 **	0.062 ***	0.032 ***	0.275 ***
5. GENDER	−0.076 ***	−0.066 ***	0.062 ***	−0.012	1	0.208 ***	0.031 ***	0.105 ***	0.013
6. AGE	0.106 ***	−0.093 ***	0.117 ***	−0.018	−0.172 ***	1	−0.330 ***	0.210 ***	−0.005
7. WORKLOAD	0.119 ***	0.013	0.079 ***	0.072 ***	0.035 ***	−0.281 ***	1	0.084 ***	0.063 ***
8. EXPERIENCE	0.009	−0.066 ***	0.130 ***	0.040 ***	0.088 ***	0.150 ***	0.114 ***	1	0.045 ***
9. EDUCATION	0.129 ***	−0.038 ***	0.301 ***	0.256 ***	0.019	0.000	0.095 ***	0.057 ***	1

The lower left section shows Spearman correlation coefficients; the upper right section shows Pearson correlation coefficients. The numbers 1 to 9 represent the variables of WORK through EDUCATION. See Table 1 for variable definitions. *, **, ***: *p* < 0.1, 0.05, and 0.01, respectively.

**Table 4:** Regression analysis of factors affecting work ability.

Variables	Pred. Sign	WORK	WORK	WORK	HEALTH
		Coefficient (t Value)	Coefficient (t Value)	Coefficient (t Value)	Coefficient (t Value)
Intercept	+/−	38.88 (14.78) ***	50.83 (20.01) ***	56.85 (21.88) ***	4.75 (10.77) ***
STRESS	-		−3.42 (−14.53) ***	−4.53 (−19.05) ***	−0.88 (−22.17) ***
SOCIAL	+		0.42 (9.7) ***	0.54 (11.86) ***	0.088 (11.59) ***
HEALTH	+		1.27 (17.7) ***		
GENDER	+/−	−0.80 (−4.78) ***	−0.87 (−5.64) ***	−0.98 (−6.23) ***	−0.09 (−3.19) ***
AGE	−	−2.50 (−4.03) ***	−3.97 (−6.91) ***	−4.51 (−7.64) ***	−0.43 (−4.26) ***
WORKLOAD	+	0.55 (7.61) ***	0.45 (6.87) ***	0.48 (7.15) ***	0.03 (2.4) **
EXPERIENCE	+	0.16 (2.43) **	0.02 (0.34)	0.01 (0.23)	−0.01 (−0.49)
EDUCATION	+	0.28 (11.49) ***	0.08 (3.5) ***	0.18 (7.73) ***	0.08 (19.45) ***
YEAR EFFECTS		YES	YES	YES	YES
*N*		7083	7083	7083	7083
F Statistic		50.22 ***	170.31 ***	140.47 ***	182.82 ***
Adj. R Square		0.046	0.193	0.151	0.188

Column 1 shows the results of the regression model containing only the control variables. Column 2 shows the results of the regression model containing the variables of interest and controls. Columns 1–3 show the results of regression models (1)–(3) for mediation analysis; the dependent variable is HEALTH. *, **, ***: *p* < 0.1, 0.05, and 0.01, respectively.

**Table 5:** Identification of mediation effects.

Effect	Coefficient Value
Total Effect ($\gamma_{1}$)	−4.53
Direct Effect ($\beta_{1}$)	−3.42
Indirect Effect ($\alpha_{1}\times \beta_{3}$)	−1.11
Percent of total effect that is mediated	24.50%

**Table 6:** Regression analysis of work ability in relation to subjective social status.

Variables	Pred. Sign	Low	Moderate	High
		Coefficient (t Value)	Coefficient (t Value)	Coefficient (t Value)
Intercept	+/−	49.38 (2.72) ***	53.05 (16.5) ***	52.14 (12.74) ***
STRESS	-	−6.11 (−4.31) ***	−3.72 (−13.34) ***	−2.90 (−6.79) ***
HEALTH	+	2.47 (6.16) ***	1.34 (15.1) ***	0.96 (8.47) ***
GENDER	+/−	−1.19 (−1.05)	−0.89 (−4.37) ***	−0.87 (−3.75)
AGE	-	−1.87 (−0.45)	−3.93 (−5.4) ***	−3.48 (−3.81) ***
WORKLOAD	+	0.50 (1.16)	0.50 (5.93) ***	0.41 (4.04) ***
EXPERIENCE	+	−0.05 (−0.15)	0.06 (0.81)	−0.01 (−0.13)
EDUCATION	+	0.08 (0.54)	0.12 (4.13) ***	0.08 (1.91) *
YEAR EFFECTS		YES	YES	YES
N		296	4768	2019
F Statistic		10.29 ***	98.83 ***	36.44 ***
Adj. R Square		0.2208	0.1559	0.1365

This table shows the results of regression models examining the effects of stress on work ability in relation to social status. *, ***: *p* < 0.1 and 0.01, respectively.

**Table 7:** Identification of mediation effects for subgroups.

Effect	Low	Moderate	High
	Coefficient Value	Coefficient Value	Coefficient Value
Total Effect ($\gamma_{1}$)	−8.73	−4.95	−3.79
Direct Effect ($\beta_{1}$)	−6.11	−3.72	−2.90
Indirect Effect ($\alpha_{1}\times \beta_{3}$)	−2.62	−1.23	−0.89
Percent of total effect that is mediated	30.01%	24.85%	23.50%

**Table 8:** Regression analysis of work ability with lagged independent variables.

Variables	Pred. Sign	WORK	HEALTH
		Coefficient (t Value)	Coefficient (t Value)
Intercept	+/−	20.75 (3.10) ***	2.24 (1.97) **
LAGWORK		0.49 (11.04) ***	
LAGSTRESS	-	−0.12 (−3.06) ***	−0.07 (−10.59) ***
LAGSOCIAL	+	−0.02 (−0.22)	0.07 (4.22) ***
LAGHEALTH	+	0.53 (3.60) ***	
GENDER	+/−	−0.75 (−1.78) *	−0.15 (−1.86) *
AGE	-	−1.34 (−0.90)	−0.07 (−0.29)
WORKLOAD	+	0.16 (1.25)	0.04 (1.48)
EXPERIENCE	+	0.13 (0.94)	0.00 (0.16)
EDUCATION	+	0.07 (1.46)	0.07 (7.92) ***
YEAR EFFECTS		YES	YES
*N*		1462	1462
F Statistic		60.05 ***	44.77 ***
Adj. R Square		0.271	0.173

This table shows the results of regression models examining the effects of stress on work ability with lagged variables. Column 2 shows the results of the regression model containing variables of interest and controls. Column 3 shows the results of the regression model for mediation analysis; the dependent variable is HEALTH. *, **, ***: *p* < 0.1, 0.05, and 0.01, respectively.
